# Electroacupuncture stimulation at sub-specific acupoint and non-acupoint induced distinct brain glucose metabolism change in migraineurs: a PET-CT study

**DOI:** 10.1186/s12967-014-0351-6

**Published:** 2014-12-12

**Authors:** Mingxiao Yang, Jie Yang, Fang Zeng, Peng Liu, Zhenhong Lai, Shufang Deng, Li Fang, Wenzhong Song, Hongjun Xie, Fanrong Liang

**Affiliations:** Acupuncture and Tuina School, Chengdu University of Traditional Chinese Medicine, Chengdu, Sichuan China; Life Science Research Center, School of Life Science and Technology, Xidian University, Xi’an, Shaanxi China; Department of Acupuncture, the Third Affiliated Hospital of Zhejiang University of TCM, Hangzhou, Zhejiang China; PET-CT Center, Sichuan Provincial People’s Hospital, Chengdu, Sichuan China

**Keywords:** Acupuncture analgesia, PEC-CT, Migraine

## Abstract

**Background:**

Acupuncture has analgesic effect to most pain conditions. Many neuroimaging studies were conducted to explore acupoint specificity in pain and other condition, but till now there is still discrepancy. Based on our previous finding, this study investigated the brain metabolism changes of acupuncture analgesia induced by sub-specific acupoint and non-acupoint stimulation.

**Methods:**

30 migraineurs were included and randomly assigned to 3 groups: Acupuncture Group (AG), Sham Acupuncture Group (SAG) and Migraine Group (MG). In AG, a combination sub-specific points of *Shaoyang* meridians, *Luxi* (TE19), *San Yangluo* (TE8), and *Xi Yangguan*(GB33) has been stimulated with electroacupuncture, while non-acupoints for SAG were used and MG received no treatment. Positron emission tomography with computed tomography (PET-CT) was used to identify differences in brain glucose metabolism between groups.

**Results:**

In the AG, brain glucose metabolism increase compared with the MG was observed in the middle frontal gyrus, postcentral gyrus, the precuneus, parahippocampus, cerebellum and middle cingulate cortex (MCC), and decrease were observed in the left hemisphere of Middle Temporal Cortex (MTC).In the SAG, compared with MG, glucose metabolism increased in the poster cingulate cortex (PCC), insula, inferior temporal gyrus, MTC, superior temporal gyrus, postcentral gyrus, fusiform, inferior parietal lobe, superior parietal lobe, supramarginal gyrus, middle occipital lobe, angular and precuneus; while, decreased in cerebellum, parahippocampus.

**Conclusions:**

Acupuncture stimulation at both sub-specific acupoint and non-acupoint yields ameliorating effect to migraine pain, but with evidently differed central mechanism as measured by PET-CT. The pattern of brain glucose metabolism change in acupoint is pertinent and targeted, while in non-acupoint that was disordered and randomized. These finding may provide new perspectives into the validation of acupoint specificity, optimizing acupuncture analgesia and revealing central mechanism of acupuncture analgesia by neuroimaging measurement.

**Trial registration:**

This trial was registered in the Chinese Clinical Trial Registry, with registration no. ChiCTR-TRC-11001813.

## Background

Acupuncture is an essential component of oriental medicine that has been persistently and extensively practiced to treat various diseases, pain in particular, in China and many other Asian countries. Doctors and patients in West countries usually employ acupuncture as adjunct to conventional drugs for a wide spectrum of pain conditions. Though it was dominantly mediated by 5-HT and endogenous opioid system, the mechanism of acupuncture analgesia remains to be elucidated.

In the long history of clinical practice, traditional acupuncture formed its therapeutic principles, and the experience of acupuncturists successively enriched the fruitfulness of acupuncture theories. According to traditional beliefs, acupuncture point, or termed acupoint, refers to specific point on the body surface that is inactive when the body is in healthy condition, but can be further activated by certain stimulus, such as needle penetration and manipulation, pressing, electrical current, heat. Well-defined acupoints selection principles for practitioners to consider in clinical situation were developed and utilized under the umbrella of the existence of acupoint specificity. However, several recent systematic reviews and clinical randomized controlled trials reported outcome that there’s minimal, or no superiority in function of acupoint compared with sham acupoint [1-5], which is incompatible with classic acupuncture theory. So that it raised questions and doubts to the existence of acupoint specificity [6].

In recent years, neuroimaging technology has been reasonably accepted as a new approach to non-invasively characterize the central interacting mechanism of acupuncture therapy [7-11]. Integrated response of cerebro-cerebellar and limbic system has been detected by fMRI after acupuncture stimulation at a specific acupoint [12]. By means of that, a growing body of evidence has been accumulated to address the existence of acupoint specificity. Many interesting articles elicited possible central response/mechanism of acupuncture specificity were published [13-15]. Our former works also indicated that remarkable modulation on the homeostatic afferent network, including the insula, anterior cingulate cortex(ACC), and hypothalamus, might be the specific mechanism of acupuncture specificity [16]. While, it is necessary to point out that the preponderance of previous studies were undergone within healthy subjects. Since the function of acupoint is body-condition dependent according to Traditional Chinese Medicine(TCM) perspectives [17], there’s a paucity of relative studies that compares the influence of acupoint on brain activity of diseased condition with that of sham acupoint. Therefore, the traits and influence of acupoint specificity for acupuncture analgesia in diseased condition remain unclear and worth investigation.

Migraine without aura is a disabling disease characterized by hemispheric headache. Current evidence favors to include acupuncture therapy in migraine care for that acupuncture yielded relatively equivalent effect to positive drugs, but there’s little side effects [18-20]. The curative function of acupuncture for migraine is not limited to instant cessation for acute pain [21,22], studies also demonstrated that acupuncture can reduce frequencies of migraine attacks as the prophylactic drugs do [23,24]. Hence, migraine is considered as a proper indication of acupuncture analgesia and is frequently chosen as a carrier to reflect the specificity or mechanism of function of acupoint. Hence, in this study migraineurs were enrolled to further investigate acupoint specificity in acupuncture analgesia.

In a previous study, we found that purposeful selection of specific acupoints on *Shaoyang* meridians, which were believed according to TCM theory to be closely related to and also are commonly applied in clinical practice to migraine treatment, presented preferred instant analgesic effect compared to those on *Yangming* meridians [25]. Meanwhile, results of PET-CT demonstrated that pain or migraine related brain regions such as MCC, and several parts of the limbic system, have been observed with more extensive metabolism change after stimulation at specific acupoints on disease dependent meridians compared with that of disease independent meridians, which implied the specificity of central responses. Secondary to this, we conducted the present study to explore whether there is difference of cerebral response after acupuncture stimulation at sub-specific acupoint on *Shaoyang* meridian, while compared to non-acupoint. The sub-specific acupoint is a concept relative to the specific acupoint, which means that its function is not so potent or focused as compared to that of the specific one. Specific acupoint is defined as points that situated in meridian line with the strongest and the most concentrated power for certain disease. The classification of specific acupoint is mostly based on traditional acupuncture theory. In contrast, non-acupoint is some random point that is absent from meridian line, and thus according to its common application it is always referred to as sham point that possesses no specific action [26,27]. Therefore, this study as a continuum was performed on the basis of previous successful application of PET-CT paradigm in migraine to indicate central response, and this is also a deepened exploration to the previous findings.

Migraine patients were recruited as voluntary subjects, which is identical to former study to observe subject in diseased condition. Also, fluorodeoxyglucose positron emission tomography combined with computed tomography (FDG-PET/CT) was main tool to visualize glucose metabolism of diverse brain regions in migraine patients. By trial designed like this, we expected to observe the difference of cerebral response in relevant groups in order to further investigate mechanism of acupuncture analgesia and acupoint specificity.

## Materials and methods

### Ethical approval

The whole research protocol had been systematically reviewed and further approved by the ethics committee of the Teaching Hospital of Chengdu University of Traditional Chinese Medicine. The approval identifier is 2007KL-001. Before the commencement of the trial, each subject was clearly explained about the trial procedures and participants that were included in this trial provided with written informed consent.

### Subjects and experimental paradigm

During the period of April 2008 to December 2009, we have included thirty right-handed patients with acute migraine without aura. The migraine patients (12 males and 18 Females; mean age 33.28 ± 8.03 years) were randomized into three groups in a 1:1:1 ratio. Patient assigned to Acupuncture at sub-specific acupoint Group (AG) received stimulation at sub-specific acupoints on *Shaoyang* meridians; Sham Acupuncture at non-acupoint Group (SAG) will conduct stimulation at non-acupoints which were located neither at acupoint site nor in meridian lines; and Migraineur Wait-List control Group (MG) as a blank control will deliver no treatment to participants. After the completion of PET-CT scan, patients in SAG and MG were given traffic compensation fee for their contributions, and also free acupuncture treatments were provided as required. Subjects were matched by gender, age, handedness, and education.

### Inclusion criteria

Eligible participant met all the following criterion were included: (1) diagnosed as acute migraine without aura according to the classification criteria of the International Headache Society for the diagnosis of migraine without aura; (2) unilateral headache in the left hemisphere; (3) Frequency of migraine attacks no less that once per month during the last three months before inclusion; (4) 2 ≤ migraine pain at recruitment and before scanning ≤ 8 as measured by Visual Analogue Scale (VAS) with terminal descriptors of “no pain” and “worst pain possible” in a range between 0 and 10; (5) duration of migraine attack no more than 24 hours to the beginning of the scan; (6) aged 20–45 years; (7) no other neurological disorders assessed by normal skull CT or MRI; (8) rescue medication should be avoided within 24 hours since the onset of the acute attack; (9)Fully agreed with all trial procedures and returned written informed consent.

### Exclusion criteria

Patients were excluded if any one of the following exclusion criterion was matched: (1) headaches that were secondary to organic disorders other than migraine, such as cerebropathy, vascular malformation, hypertension, or arteriosclerosis; (2) other conditions like psychosis, bleeding disorders, or allergies; (3) other pain conditions that may contaminate the nociceptive sensation of migraine; (4) included in other study; (5) pregnancy or lactating, or participants considering to conceive in the following 6 months; (6) medication with vasoactive agent in the last two weeks; (7) with emotional disorders such as major anxiety disorder and/or depression; and (8) presence of any contraindications to PET-CT or electroacupuncture.

### Interventions

**Figure 1:**
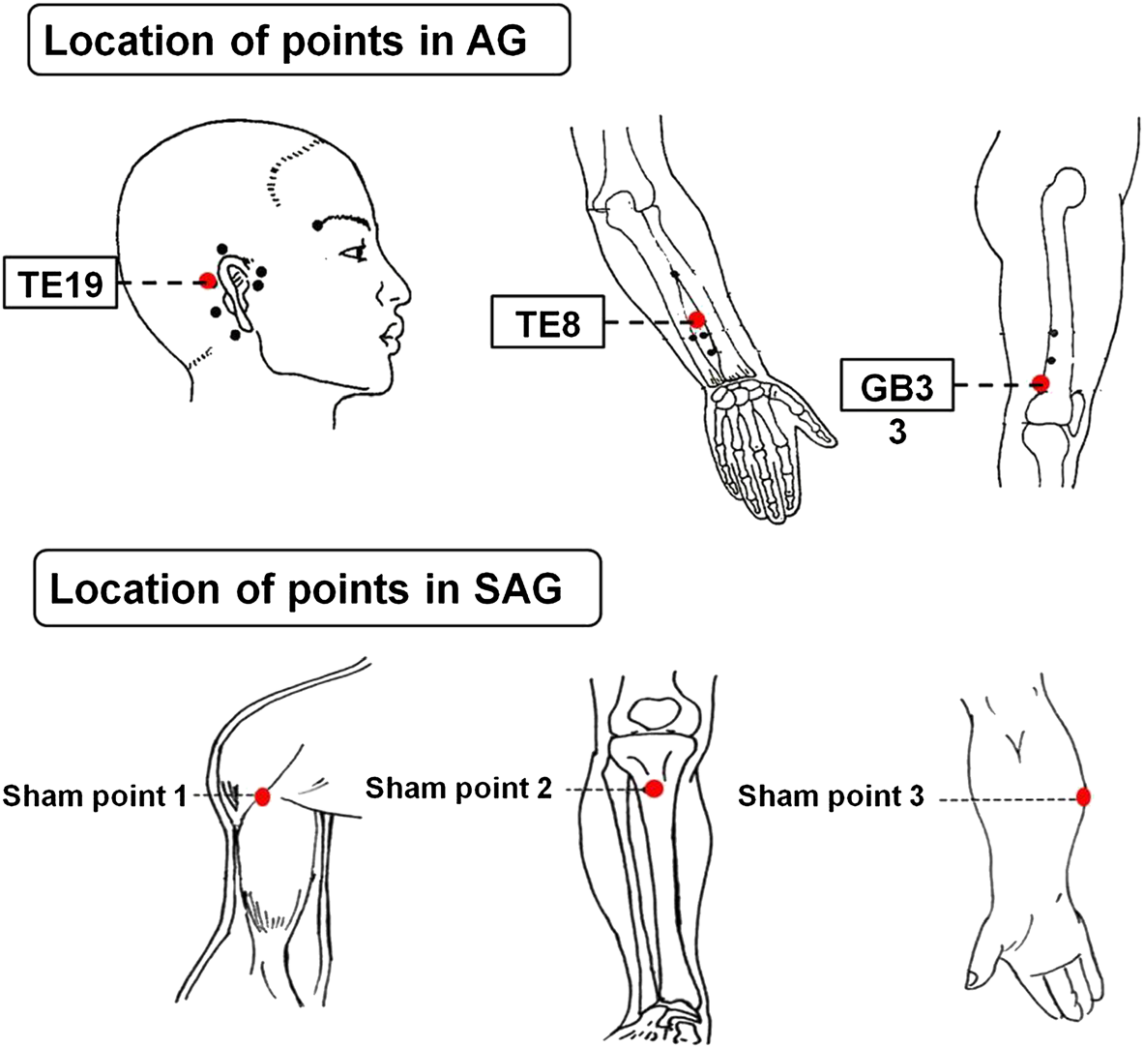
**Location of points in AG and SAG.**

Included participants were averagely assigned to either one of the three groups by means of simple randomization. The acupoint combination for the AG included three sub-specific acupoints on *Shaoyang* meridians, *Luxi* (TE19), *San Yangluo* (TE8), and *Xi Yangguan* (GB33). Non-acupoints applied in the SAG were predefined and utilized in previous studies of our team [21]. The locations of these non-acupoints were described as follows: (1) Sham-point 1:The medial side of the arm at the anterior border of the insertion of the deltoid muscle at the junction of the deltoid and biceps muscles; (2) Sham-point 2:The edge of the tibia ,1–2 cm lateral and horizontal to the *Zusanli*, ST36; (3) Sham-point 3: On the ulnar side of the arm, half way between the epicondylus medialis of the humerus and the ulnar side of the wrist. These three points were sited distant to the traditionally recognized acupoints or meridians lines. Illustrations of these points were showed in Figure 1. Only points on the left side of the body were used in the experiment.

After sterilization in local dermal area of sub-specific acupoint/non-acupoint, sterile disposable acupuncture needles (25–40 mm in length and 0.30 mm in diameter; Hwato acupuncture needles manufactured by Suzhou Medical Supplies Co., Ltd., Suzhou, China) were inserted transversely or obliquely into to the points to a depth of 15–30 mm. After needle insertion, in AG needles were evenly twirled and rotated to induce the *DeQi* sensation(soreness, numbness, distention, heaviness, dull pain, etc.) [28,29]. After the *DeQi* phenomenon was achieved and reported by patient, auxiliary needles were perpendicularly punctured 2 mm lateral to the points, to 2 mm in depth, without manual manipulation. Electrodes of Han’s acupoint nerve stimulator (HANS; model LH 200A; TENS, Nanjing, China) were then connected to the needles by acupuncturist. While, in SAG HANS was connected to needles after insertion and required no *DeQi* sensation elicited. The frequency of stimulation was 100 Hz, and the intensity of the electricity stimulus varied from 0.1 to 1.0 mA, which was manually adjusted according to when a comfortable sensation was induced to patient. The needles that are connected with electrical nerve stimulator were retained in body for 30 min. The MG was left blank with no intervention.

### PET-CT imaging

**Figure 2:**
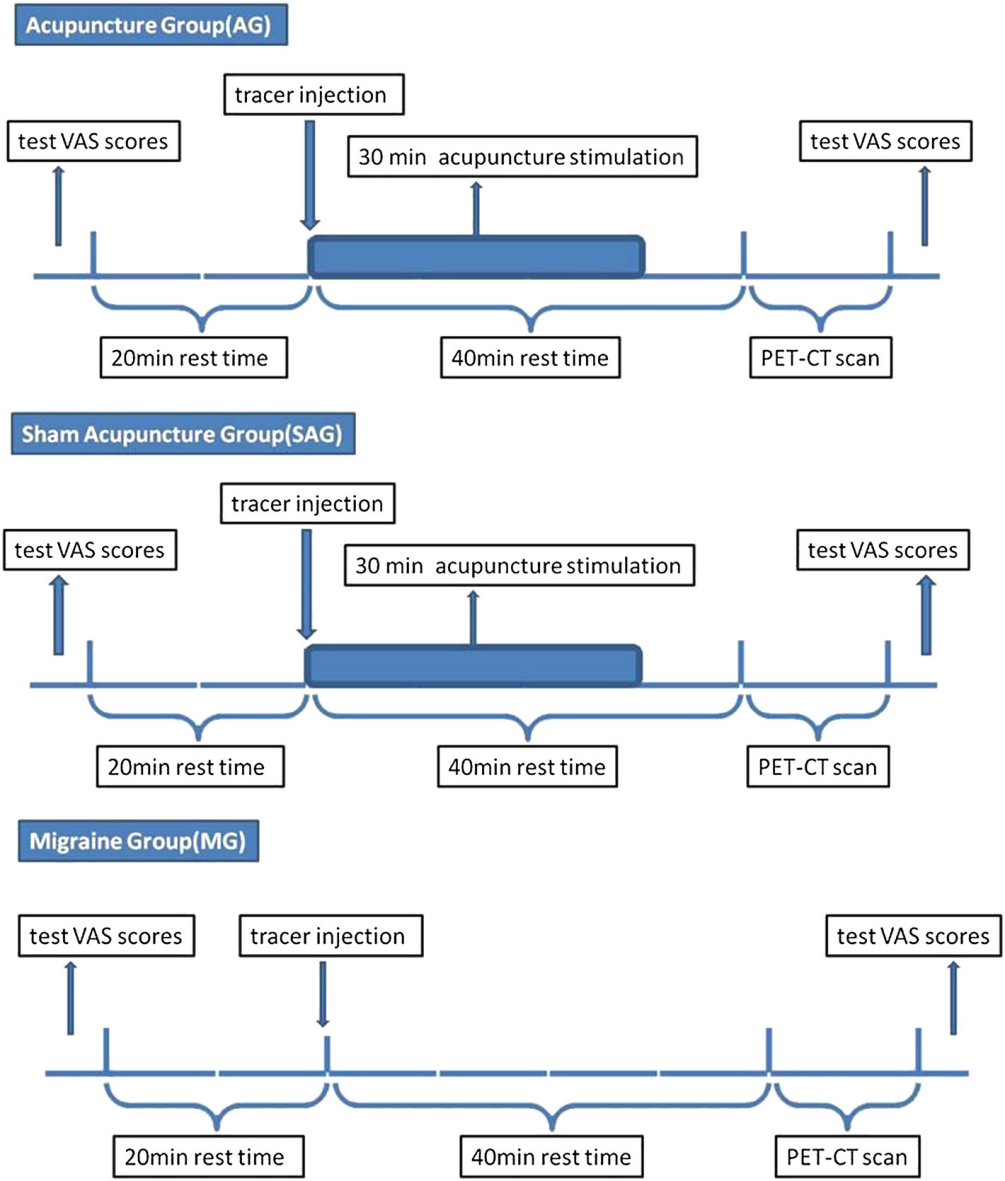
**Experimental Paradigm.** PET-CT scans were performed on the subjects at the PET-CT center of the Sichuan Provincial People’s Hospital. When the migraine attack began, each subject went through the following procedure(Figure 2): (1) examinations of blood sugar, blood pressure and Visual Analogue Scale (VAS) scores (range from 0 to 10) before the PET-CT scan; (2) a 20 min rest in a quiet, dim room; (3) a tracer injection at the back of the right hand (18F-FDG; synthesized with Mini Tracer accelerator; 0.11 mCi/kg dosage); (4) a 40 min rest, which included the 30 min acupuncture treatment in the AG and SAG; (5) a PET-CT scan; and (6) examination of VAS scores after the PET-CT scan (Figure 2). Subjects were instructed to remain relaxed during the whole study, with eyes blindfolded and ears plugged.

PET-CT scanning was performed in the PET-CT Center of Sichuan Provincial People’s Hospital, Chengdu, Sichuan, China. All patients with acute migraine attack were required to fast for at least 4 hours before PET-CT scan. Before all trials procedure, fasting plasma glucose and resting blood pressure were examined prior to acupuncture treatment and PET-CT scan to avoid faint. PET-CT imaging data sets were acquired by using a Biograph Duo BGO scanner (Siemens, Germany). Our previous attempt to characterize central response of acupuncture by using PET-CT suggested that in migraine patients the curative effect of acupuncture in brain could be responsively reflected by neuroimaging tools such as PET-CT approximately 30 to 40 min since the initiation of acupuncture stimulation [25]. Moreover, according to the guidelines of the European Association of Nuclear Medicine Neuroimaging Committee (ENC)For PET brain imaging using [^18^F]FDG, version 2 [30], it is recommended that for interventions the paradigm usually start at the time of injection and have to be maintained for a time period of at least 15–20 min [31,32]. Thus, the acupuncture treatment in this study started immediately after the injection of tracer and maintained for 30 min. The ENC also suggested that the acquisition should not start earlier than 30 min after the tracer ([^18^F]-FDG with a half-time of 109 min [33]) injection, and commonly the time frame of 30 to 60 min after tracer injection was recommended to acquire PET-CT data set. Therefore the PET-CT data set was acquired 40 min after the tracer injection, aiming to elucidate the dynamic change of brain metabolism after acupuncture. The pain severity of migraine was measured by VAS before and after scanning, respectively. The PET-CT scanning paradigm was shown in Figure 2. The whole brain image was captured in a way to be horizontal to the AC-PC line. There was a 40 min uptake period before image acquisition(bed: 1; collection mode: 3D; slice thickness: 3 mm; slice interval: 1.5 mm; matrix size: 256 × 256; total counts: 3 × 109). The ordered-subset expectation maximization (OSEM) with 6 iterations and 16 subsets was utilized to reconstruct the images once the data acquisition was accomplished.

### Image processing

The PET-CT images were processed using SPM2 software (Welcome Department of Cognitive Neurology, University College London, UK). After realignment, the images were normalized using the Montreal Neurological Institute (MNI) template and then smoothed using a Gaussian kernel with 6 mm full width at half maximum (FWHM). On the second level of statistical analysis, the differences between the AG and MG and between the SAG and MG were tested using separate two-sample t-tests. The statistical threshold was set at P < 0.05 with correction for false-discovery rate (FDR) and the cluster size threshold was >5 voxels.

## Results

### Effect of acupuncture on pain

**Figure 3:**
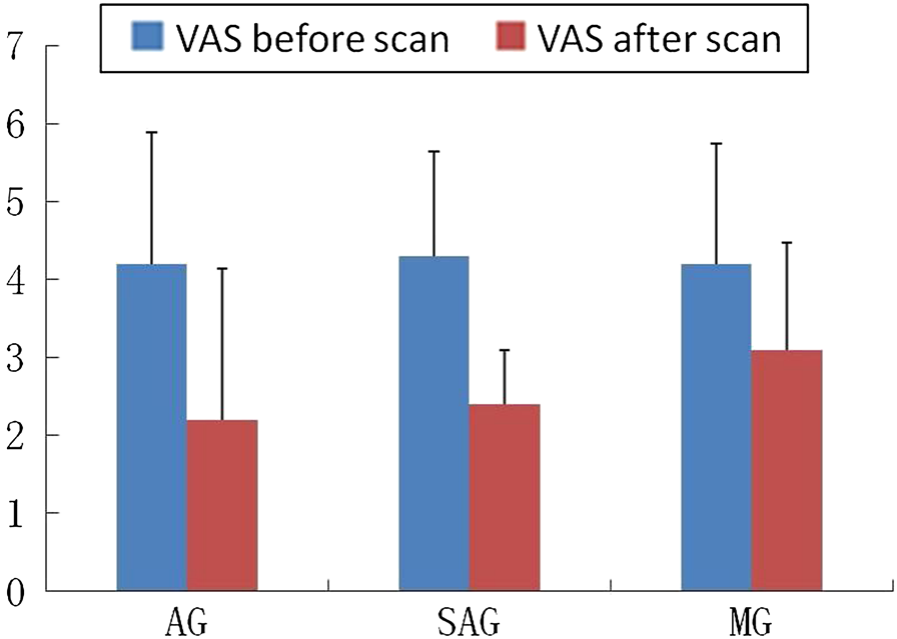
**Behavior data analysis.**

The pain intensity measured by VAS was significantly reduced in the AG (P < 0.05) and SAG (P < 0.05) after acupuncture treatment compared with baseline (paired two-tailed t-test with threshold at P < 0.05). The reduction of pain intensity appeared no significant differences between AG and SAG. There was no significant change in pain intensity in the MG (P = 0.047) (Figure 3).

## PET results

**Figure 4:**
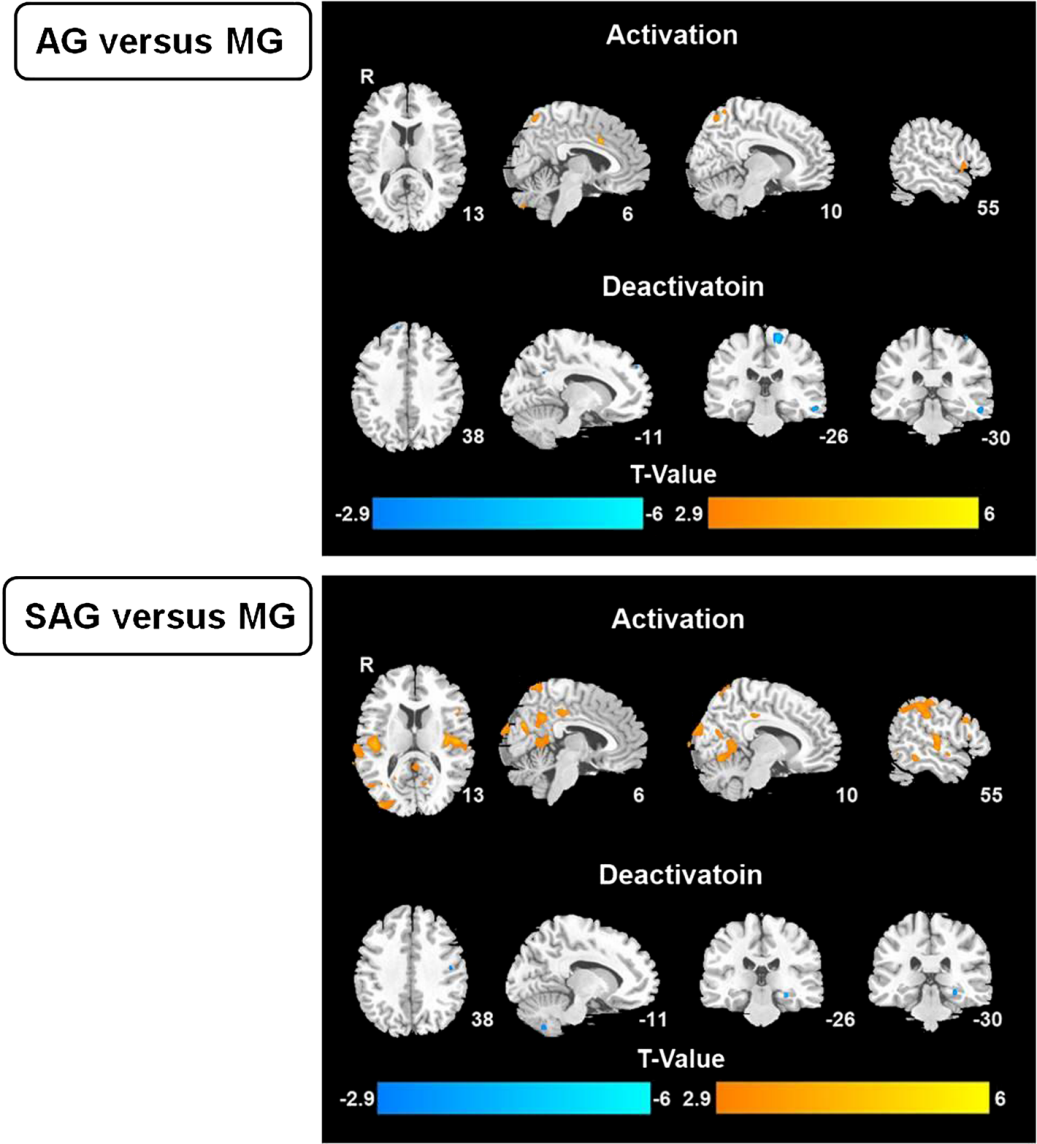
**Imagine data analysis.**

In the AG, metabolism increased compared with the MG was observed in the middle frontal gyrus, postcentral gyrus, the precuneus, parahippocampus, cerebellum and middle cingulate cortex (MCC), and decreased in the left hemisphere of Middle Temporal Cortex (MTC) (Table 1 and Figure 4). In the SAG, compared to MG, metabolism increased in the PCC, insula, inferior temporal gyrus, MTC, superior temporal gyrus, postcentral gyrus, fusiform gyrus, inferior parietal lobe, superior parietal lobe, supramarginal gyrus, middle occipital lobe, angular and precuneus, and decreased in cerebellum, parahippocampus (Table 2 and Figure 4).

**Table 1:** **Main localization of increased/decreased glucose metabolism of brain regions, comparing AG versus MG, using two-sample t-test**

**Brain region**	**Hem (L/R)**	**BA**	**Talairach**			**T-value**	**Metabolism Change (AG-MG)**
			**X**	**Y**	**Z**		
MiddleFrontal	L	6/11	−24	38	−12	5.84	↑
MiddleFrontal	R	6/11	24	−13	58	4.49	↑
Postcentral Gyrus	L	7	−12	−51	63	3.53	↑
Precuneus	L	7	−8	−61	58	4.20	↑
MCC	L	24/32	−6	13	31	4.98	↑
Parahippocampus	R	34/35	22	−13	−25	3.53	↑
Cerebellum	L	-	−6	−77	−31	3.11	↑
Middle Temporal	L	21	−59	−28	−12	−4.37	↓

Abbreviations: BA-Brodmann area; Hem-hemisphere; L-Left; R-Right; MCC-middle cingulate cortex; ↑metabolism increased; ↓metabolism decreased.

**Table 2:** **Main localization of increased/decreased glucose metabolism of brain regions, comparing SAG versus MG, using two-sample t-test**

**Regions**	**Hem (L/R)**	**BA**	**Talairach**			**T-value**	**Metabolism Change (SAG-MG)**
			**X**	**Y**	**Z**		
MCC	R	23	6	−50	36	4.531	↑
PCC	R	26	3	−40	25	3.547	↑
Insular	R	48	38	−17	12	5.234	↑
Inferior temporal	R	20	57	−42	−12	4.484	↑
	L	20	−53	−46	−14	3.406	↑
Middle temporal	R	37	47	−70	3	3.453	↑
	L	37	−62	−51	−9	4.438	↑
Superior temporal	R	48	54	−16	9	5.234	↑
	L	48	−46	−21	10	4.531	↑
Postcentral	L	3	−31	39	69	3.266	↑
Fusiform	R	18	24	−75	−14	4.203	↑
	L	37	−24	−52	−13	4.016	↑
Inferior parietal	R	40	51	−46	46	3.922	↑
	L	40	−46	−39	48	3.969	↑
Superior parietal	R	7	19	−70	63	2.75	↑
	L	7	−36	−68	56	3.266	↑
Supramarginal	R	40	60	−33	36	5.422	↑
Middle occipital	R	37	47	−72	4	3.125	↑
	L	37	−48	−68	6	5.141	↑
Angular	R	39	45	−68	47	2.512	↑
	L	39	−39	−70	52	3.688	↑
Precuneus	R	23	6	−56	29	3.359	↑
	L	23	−11	−54	27	3.549	↑
Cerebellum	R	-	40	−54	−49	−3.641	↓
	L	-	−39	59	−49	−3.781	↓
Parahippocampus	R	20	29	−26	−12	−4.578	↓
Hippocampus	R	20	30	−27	−11	−4.063	↓
Thalamus	R	-	19	−12	14	−2.375	↓

Abbreviations: BA-Brodmann area; Hem-hemisphere; L-Left; R-Right; MCC-middle cingulate cortex; PCC-poster cingulate Cortex; ↑metabolism increased; ↓metabolism decreased.

## Discussions

According to theory of TCM, the basis of acupuncture knowledge is rooted in the system of meridians and collaterals, both of them are well differentiated. So as the acupoints are categorized as specific points and sub-specific points (also known as plain points) in accordance with relevance to different indications, moreover, to a large domain as acupoint and non-acupoint. Literally, the sub-specific acupoint is a relative concept that plain acupoint showed less specificity in function compared with specific acupoint, but relatively more than non-acupoint. In previous study, we selected specific acupoints on *Shaoyang* meridian compared to *Yangming* meridian to treat acute migraine pain. Points on *Shaoyang* meridian led to specific cerebral response of MCC, MTC, MFC, OFC, insula, PCC. It is believed that MCC participate in the action of interference to attention in the process of pain [34]. Therefore, we suggested that it may symbolize possible specificity of acupoints. In particular, white paper in 2010 authorized by American Association of Acupuncture pointed out that acupoint specificity remains a paradox of acupuncture research [35]. Thus, in this study the comparison in clinical effectiveness and central response that was characterized by PET-CT provides some additive evidence to support the acupoint specificity.

Based on principles of traditional acupuncture, specific acupoints on relevant meridians have clinical superiority in contrast to that without relevance, or sub-specific acupoint. Coincidently, that’s consistent to what have been indicated in the previous study, no matter from the aspect of neuroimaging or clinical analgesic effect. Subsequent to results of former study, we still selected points on *Shaoyang* meridian, but not specific points, with comparison to non-acupoint. PET-CT was adopted to explore the difference between analgesic effects reflected by cerebral responses, aiming to further elucidate acupoint specificity. The results manifested that brain pattern of metabolism change varied from AG to SAG compared to MG, while difference of existed the analgesic effect may not be yielded. Specifically, from the behavioral level, analgesia existed in both groups, but with no significant difference between AG and SAG. However, as a result of comparatively short observing period and a lack of subjects, it requires further clinical trials with longer fellow-ups to draw a conclusion.

According to several clinical RCTs, acupuncture stimulation at verum acupoint as well as sham acupoint can reduce instant pain for migraine attacks [21,22].Through analysis of our clinical data, decreased of VAS has been obtained after acupuncture in both AG and SAG. This maybe simply indicates that the penetration of needles into the skin may induce analgesic effect and benefit migraine patients in terms of somatic pain. Moreover, quantitive reduction of VAS in AG is slightly superior to SAG, but the difference between them shows no statistically significance. The slight superiority may not be sufficient for us to conclude which one is superior or equivalent to another in terms of instant analgesia generated by acupuncture stimulation. Besides, this trial enrolled 30 migraine patients totally with 10 to every single group. Whereas the limitation of subject quantity and minor clinical superiority, it is still uncertain to whether acupoint functions better than sham-acupoint.

In fact, the results of visualized glucose metabolism change in responded brain regions are far more interesting. Despite the fact that the central mechanism of analgesia induced by acupuncture in verum and sham acupoint is still unclear, sizeable studies with methods of neuroimaging have proposed theoretical potentialities in several pain related brain regains [36,37]. As to current study, both groups showed increase of glucose metabolism in brain regions of the Postcentral Gyrus, the precuneus and middle cingulate cortex (MCC). There’s decrease of metabolism for AG and SAG in left hemisphere of Middle Temporal Cortex (MTC) and Cerebellum, Parahippocampus, respectively. These interacted brain regions are very similar to those reported in our previous paper in which *Shaoyang* meridians were considered as the most relevant channels to migraine and has been stimulated. Furthermore, MCC has been reported to be relevant to negative emotions and motor signals [38]. Besides, it is well known that insula is closely related to pain, attention, emotion, and visual input, especially in migraine [39,40]. However, in current study no metabolism change of insula was observed. For this, it is speculated as a difference in central responses between specific and sub-specific acupoint.

Elsewise, it presented activation of more extensive brain regions in SAG compared to AG, including PCC, insula, inferior temporal gyrus, MTC, superior temporal gyrus, fusiform gyrus, inferior parietal lobe, superior parietal lobe, supramarginal gyrus, middle occipital lobe and angular. To our knowledge, some of the above regions have already been reported as pain or migraine related area, but some of them not. Although we cannot dispel any further possibility of relationship of those brain regions to pain, it is still possible that SAG may generally induce brain responses without specifically targeted site and presented a random and non-specific brain response, as shown in this study.

## Conclusions

This study demonstrated that acupuncture stimulation at both sub-specific acupoint and non-acupoint produces ameliorating effect to migraine pain, but with evidently differed central mechanism as measured by PET-CT. The pattern of brain glucose metabolism change in acupoint is pertinent and targeted; while in non-acupoint its distribution was disordered and randomized. These finding may provide new perspectives into the validation of acupoint specificity and central mechanism of acupuncture analgesia by neuroimaging measurement. Most of all, we believe that the identification of superb acupoint for analgesia by comparing various class of acupoint is surely helpful to the optimization of acupoint selection in clinical practice. To further validate the hypothesis and explore the mechanism of acupoint specificity, more investigations are required.

## Abbreviations

AG: Acupuncture Group
SAG: Sham Acupuncture Group
MG: Migraine Group
PET-CT: Positron emission tomography with computed tomography
FDG-PET/CT: Fluorodeoxyglucose positron emission tomography combined with computed tomography
MCC: Middle cingulate cortex
MTC: Middle Temporal Cortex
PCC: Poster cingulate cortex
ACC: Anterior cingulate cortex
TCM: Traditional Chinese Medicine
HANS: Han’s acupoint nerve stimulator
OSEM: Ordered-subset expectation maximization
MNI: Montreal Neurological Institute
FWHM: Full width at half maximum
FDR: False-discovery rate
VAS: Visual Analogue Scale
